# Association between Kinesiophobia and Gait Asymmetry after ACL Reconstruction: Implications for Prevention of Reinjury

**DOI:** 10.3390/ijerph18063264

**Published:** 2021-03-22

**Authors:** Hossein Tajdini, Amir Letafatkar, Britton W. Brewer, Mahdi Hosseinzadeh

**Affiliations:** 1 Department of Biomechanics and Sport Injury, Faculty of Physical Education and Sports Sciences, Kharazmi University, Tehran 1571914911, Iran; h_tajdini@yahoo.com (H.T.); letafatkaramir@yahoo.com (A.L.); 2 Department of Psychology, Springfield College, Springfield, MA 01109, USA; 3 Department of Sport Injuries and Corrective Exercises, Sport Sciences Research Institute, Tehran 1587958711, Iran; metti@ssrc.ac.ir

**Keywords:** prevention of sports injuries, ACL reconstruction, reinjury, kinesiophobia, gait asymmetry, psychological factors, biomechanics

## Abstract

Gait asymmetries have been documented in individuals after anterior cruciate ligament (ACL) reconstruction (ACLR). The relationship between gait asymmetry and associated psychological factors, however, is not yet known. This study aimed to examine the relationship between kinesiophobia (fear of reinjury) and asymmetry of vertical ground reaction force (vGRF) and lower-extremity muscular activity in individuals after ACLR during gait. Twenty-eight males with a history of ACLR participated in the study. Force plate and surface electromyography was used to record peak vGRF and muscular activity. The Tampa Scale of Kinesiophobia (TSK-11) was used to measure kinesiophobia. Spearman’s rank correlations analysis was used to examine the relationship between TSK-11 scores and both gait asymmetry variables. There was a significant positive relationship between TSK-11 and asymmetry of the second peak of vGRF (*r_s_* = 0.531, *p* = 0.002). In addition, there was a significant positive association between asymmetry of rectus femoris activity (*r_s_* = 0.460, *p* = 0.007) and biceps femoris activity (*r_s_* = 0.429, *p* = 0.011) in the contact phase. Results revealed a significant relationship between kinesiophobia and asymmetry in muscle activity and vGRF in different phases of the gait cycle. Interventions addressing kinesiophobia early in the rehabilitation after ACLR may support the restoration of gait symmetry, facilitate a more rapid return to sport, and reduce the risk of ACL reinjury.

## 1. Introduction

Even with innovative surgical and rehabilitation approaches, previous studies have reported a high rate of anterior cruciate ligament (ACL) reinjury and fast-developing knee osteoarthritis (OA) after ACL reconstruction (ACLR). Previous studies have shown that up 23% of patients with ACLR had ACL reinjury [1], and the prevalence of radiographic knee OA of patients with combined ACL and meniscal injuries at 10 to 15 years follow-up after ACLR was 80% [2]. In addition, young athletes who return to sport are at approximately 30 to 40 times greater risk of sustaining an ACL injury during sport, relative to their uninjured counterparts [1].

Gait asymmetries following ACLR are evident at 6 months after surgery [3,4] and can persist for many years [5,6]. Asymmetry of lower-limb movements may contribute to the development of OA and increase the risk for reinjury in people with ACL-injured knees [6,7,8]. The first year after ACLR is a critical period for athletes who try to regain their preinjury level of functioning, and the likelihood of experiencing another knee injury is around 49% [9]. Previous research indicates that athletes with biomechanical asymmetries at the time of return to sport (RTS) were at least three times more likely to reinjure their ACL within one year compared to those without asymmetries [8]. Asymmetrical lower-extremity loading also alters chondrocyte synthesis and catabolic activities, which can lead to structural damage of articular cartilage and may accelerate the development of knee OA [10,11]. People who have had ACLR experience changes in matrix densities as early as 1 year following initial injury [12] and are at increased risk for joint degeneration and post-traumatic OA [13]. In a recent study, asymmetrical loading during gait shortly after ACL injury and six months after ACLR were associated with radiographic symptoms of OA 5 years after surgery [14]. Higher lower-extremity loading during gait is likely more meaningful for cartilage health given the quantity of steps taken per day relative to the average daily volume of more intense physical activity.

Reductions in quadriceps femoris muscle strength after ACLR are also common [15,16], but prior literature is mixed in terms of their relationship to gait asymmetry, indicating that other biomechanical or contextual factors are likely involved [17,18,19,20]. For instance, psychological factors could contribute to gait asymmetry after ACLR [20]. Research has addressed various psychological factors such as fear of movement, fear of reinjury, confidence, psychological readiness to return to sport (RTS), and self-efficacy [20,21,22,23,24,25]. Kinesiophobia has been examined in other musculoskeletal disorders as well, such as hallux valgus [26]. Fear of movement and reinjury tends to decrease during postoperative rehabilitation, and is associated with knee function at a time when athletes typically RTS [21]. Additionally, changes in self-efficacy and fear of movement/reinjury for rehabilitation tasks predict change in function during postoperative rehabilitation [27], and psychological factors such as psychological readiness to return to sport, fear of reinjury, sport locus of control [22], and motivation during rehabilitation [24] are associated with RTS outcomes after ACLR.

Fear is a prominent psychological response at the time that athletes resume sport participation after injury [28]. In addition, fear of reinjury is one of the most frequently cited reasons for not returning to sport after ACLR [29]. In previous research, athletes who did not RTS after had higher mean scores on the Tampa Scale of Kinesiophobia-11 (TSK-11) than those who did RTS after ACLR [23]. The TSK has been used to explore fear of reinjury before, immediately after, and up to 20 years after ACLR [21,23,25,27,30,31,32]. Relative to patients with low fear of reinjury, those with elevated fear of reinjury have lower rates of RTS [21,23,29,30,33] and self-reported activity [32], lower self-reported functioning of knee [21,31], lower-quadriceps strength [23,32], and increased risk of reinjury [32].

Therefore, early attention to psychological factors in individuals after ACLR is warranted but requires further research. Specifically, there is an empirical void regarding the potential association between psychological factors and gait asymmetry. Therefore, we aimed to examine the connection between kinesiophobia (fear of reinjury) and gait asymmetry after approximately six months of rehabilitation following ACLR. We hypothesized that greater fear of reinjury of athletes with ACLR would be associated with greater asymmetry in lower-limb muscle activity and vertical ground reaction force (vGRF).

## 2. Materials and Methods

### 2.1. Study Participants

Twenty-eight males (n = 28) who received unilateral ACLR using hamstring tendon autograft within ≤6 months prior to the study (age = 23.7 ± 2.1 years, height = 179.2 ± 8.5 cm, weight = 78.1 ± 7.9 kg, time from ACLR to testing = 173.2 ± 6.2 days) volunteered to participate for this cross-sectional investigation. All participants played a sport involving cutting, pivoting and/or jumping before their injury (at least three times per week for 30 min per session). All participants sustained isolated ACL injuries with no damage to ligaments, menisci, cartilage, or bone. All participants performed similar postoperative home-based rehabilitation programs, under the supervision of a physical therapist, and had normal knee motion range while conducting the study. The participants’ mean International Knee Documentation Committee (IKDC) score was 62.35 ± 10.72. This study was approved by the Sport Science Research Center Institutional Review Board.

### 2.2. Procedure

Participants were invited to the laboratory for two sessions: a familiarization session and a testing session. During the familiarization session, participants signed a consent form, provided demographic, physical activity, and injury history data, and completed the TSK-11 [34,35]. The testing and measurement process was explained to the participants. At the end of the familiarization session, participants were instructed to return to the laboratory within 48 h to complete their test session. The TSK-11 was used to measure the fear of reinjury in athletes after ACLR. The TSK-11 is an 11-item questionnaire that scales each question from 0 to 4 (Likert scale). The total score is calculated by adding up responses to the 11 items in which the possible scores ranged from 11 to 44. Greater scores on the TSK-11 show higher fear of reinjury. The internal consistency of the TSK-11 is acceptable (Cronbach’s *α* = 0.79) and also its test–retest reliability (SEM = 2.54, ICC = 0.81) [34].

After entering the laboratory for the testing session, participants performed 5 min of dynamic warm-up, which included gait swings, light jogging, and plyometrics. After shaving and cleansing the skin, surface Ag–AgCl electrodes (Australia’s SKINTACT model) were placed, with an inter-electrode distance of 2 cm, longitudinally over the following muscles in both injured and uninjured limbs according to the European SENIAM protocol [36]: rectus femoris, biceps femoris, and medial gastrocnemius. Participants performed three repetitions for 5 s each of a maximum voluntary isometric contraction (MVC) for the rectus femoris, biceps femoris, and medial gastrocnemius during manual muscle tests. A one-minute rest period was given between the MVC trials.

Next, participants were instructed to walk barefoot and were given up to three practice trials. Immediately afterward, participants performed three walking tasks (the rest interval between trials was one minute) during which the data were recorded. Successful trials required that participants land a clean foot-strike onto the force plates (Bertec, 40 ×60, USA) while walking at a self-selected pace and without an awareness of the position of the force plates. All trials were performed along an 8-m walkway (approximately 4.5 m prior to force plate contact). Quadriceps strength was measured after the walking test.

### 2.3. Data Analysis and Reduction

Ground reaction forces (GRF) data were collected using force plates with a sampling frequency of 500 Hz. GRF data were filtered using a fourth-order, zero-lag, low-pass Butterworth filter with a 20 Hz cut-off frequency [4]. The chosen threshold for ground contact was defined as the time when vertical GRF (vGRF) exceeds 10 N. Peak vGRF were calculated during the walking for each subject trial, and normalized to the subject’s body weight (%BW). For vGRF, we extracted two peaks: the maximum of the first half (first peak) and the maximum of the last half (second peak) during stance phase. These vGRF values were extracted for both injured and uninjured limb.

The surface electromyography (EMG) data were measured using EMG system (MT8 Model, MIE, UK) synchronized with the force plates. The EMG data were collected at 1000 Hz and filtered using a 10–450 Hz fourth-order band pass Butterworth filter and were smoothed using a 50 ms moving window root mean square (RMS) algorithm [4]. All of the trials were normalized to 100% of stance. RMS activity index was used to calculate the amount of electromyography activity of the muscles during walking, during the contact phase (the first 25% of the stance phase) and the combined midstance/propulsion phase (the latest 75% of the stance phase). To normalize the EMG data, the values obtained from the RMS were divided by the values obtained from the MVC of each muscle and are reported as a percentage of the MVC.

Quadriceps isometric strength was measured using an isokinetic dynamometer (Biodex System 3; Biodex) during a MVC while sitting with 90° hip flexion and 60° knee flexion. The lateral femoral condyle was accorded to the rotation axis of the dynamometer, and the dynamometer resistance pad was secured to the anterior dimension of the distal shank. Participants were asked to perform submaximal practice to familiarize themselves with the device. After getting acquainted with the device, the subjects were asked to perform three trials with maximum effort (within 5 s, 60 seconds’ rest between trials) for each limb. Quadriceps strength was normalized to the athlete’s mass (kg), resulting in Nm/kg.

All data processing occurred in MATLAB R2016a software (The MathWorks, Inc., Natick, MA, USA). A formula developed by Robinson et al. was used to obtain the asymmetry indices [37].

$$\mathrm{Asymmetry}\;\mathrm{index}=\frac{\left(X_{i}-X_{u}\right)}{0.5(X_{i}+X_{u})}\times 100$$

where *X_i_* and *X_u_* are the values of the specified parameter for the injured and uninjured limbs. Therefore, a positive asymmetry would be indicative of a higher value for the injured limb.

### 2.4. Statistical Analysis

The Shapiro–Wilk test was employed to assess the normal distribution of data. A paired *t*-test was used to compare the quadriceps isometric strength between the injured and uninjured limbs. Spearman Rank tests (*r*_s_) were used to examine the relationship between the scores from the TSK-11 and asymmetry in vGRF and muscle activity. The associations were interpreted as: nearly perfect (*r* = 0.90–1.00), very high (*r* = 0.70–0.89), high (*r* = 0.50–0.69), moderate (*r* = 0.30–0.49), low (*r* = 0.10–0.29), and trivial (*r* < 0.10) [38]. All statistical analyses were run using SPSS software version 22. In this study, a significance level of *p* < 0.05 was employed.

## 3. Results

**Figure 1:**
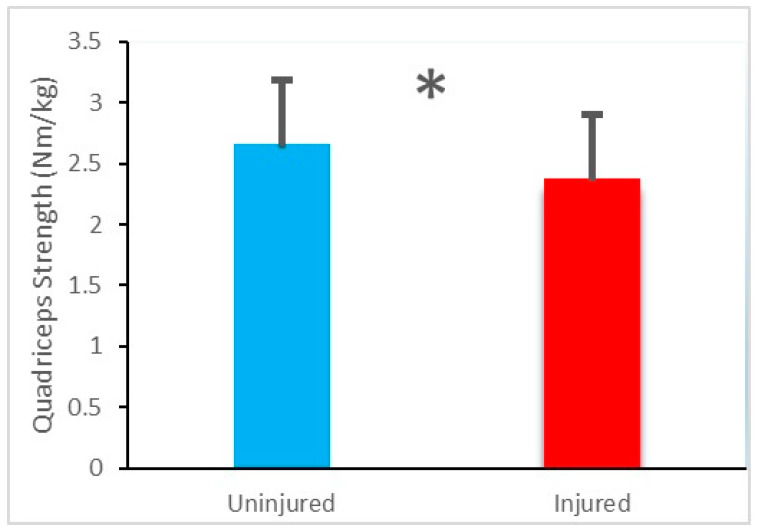
Mean and standard deviation values of quadriceps strength of the injured limb and uninjured limb. * Denotes a significant difference.

The quadriceps strength of the injured limb (2.38 ± 0.50 Nm/kg) was significantly lower than that of the uninjured limb (2.66 ± 0.54 Nm/kg), *t*(27) = 11.82, *p* < 0.001 (Figure 1).

The average TSK-11 score for the athletes with ACLR was 22.67 ± 4.63. Means and standard deviations of vGRF and muscle activity in injured and uninjured limbs are presented in Table 1.

The relationships between TSK-11 scores and asymmetry of vGRF variables while walking is shown in Table 2, which indicates that there was a positive and significant relationship between TSK-11 scores and asymmetry of the second peak of vGRF (*r*_s_ = 0.531, *p* = 0.002). There was, however, no significant relationship between TSK-11 score and asymmetry of the first peak of vGRF (*p* > 0.05).

The relationships between TSK-11 scores and the asymmetry of muscle activity while walking indices are displayed Table 3, which indicates that there was a moderate positive relationship between greater TSK-11 scores and greater asymmetry of rectus femoris activity (*r*_s_ = 0.460, *p* = 0.007) and biceps femoris activity (*r*_s_ = 0.429, *p* = 0.011) in the contact phase. Also, there was no significant relationship between TSK-11 scores and asymmetry of medial gastrocnemius activity in the contact phase and asymmetry of medial gastrocnemius, rectus femoris, and biceps femoris activity in the midstance/propulsion phase (*p* > 0.05).

## 4. Discussion

This study aimed to determine the association between kinesiophobia (fear of reinjury) and asymmetry in vGRF and lower-limb muscle activity during walking in individuals with ACLR. The findings support our hypothesis that fear of reinjury would be positively correlated with asymmetry of the second peak of vGRF and asymmetry of rectus femoris activity and biceps femoris activity in the contact phase. We also found that the asymmetry of medial gastrocnemius activity in the contact phase, the asymmetry of medial gastrocnemius, rectus femoris, and biceps femoris activity in the midstance/propulsion phase, and the asymmetry of the first peak of vGRF were not related to fear of reinjury. The presence of asymmetry in human gait causes more stress on one limb than other limbs and reduces the function of the other limb.

Compensation by the contralateral limb or bilateral deficits may influence the comparison between the involved limb and the contralateral limb during walking after ACLR. Indeed, bilateral deficits have been documented after ACL rupture and/or reconstruction [39,40,41], and bilateral alterations (injured limb and the contralateral limb) as compared to control while walking after ACL-R [3,4,42,43] have been reported up to 2 years after ACLR [6,9,44,45,46]. Nonetheless, movement asymmetries accompany both the development of osteoarthritis [2,14] and the likelihood of a second calamitous injury [8,9]. Athletes with asymmetries at the hip and knee 1 year after ACLR are at least 3 times more probable to experience a second ACL injury than those without asymmetries [8]. Asymmetrical lower-extremity loading may also contribute to the development or progression of post-traumatic knee OA, a disease found in almost half of those with ACLR within 10 to 15 years after surgery [2]. It was found in a recent study that asymmetrical loading (i.e., underloading the surgical limb) during gait early after ACL injury and 6 months after ACLR were associated with radiographic signs of OA 5 years after surgery [14]. The continuation of such anomalies during tasks as simple as walking may pose serious dangers to the long-term well-being of the injured joint.

The increase in asymmetry in the second peak of vGRF in patients with ACLR was due to a decrease in this force in the injured limb. Also, greater asymmetry resulted from an increase in the injured limb’s rectus femoris activity and biceps femoris activity in contact phase. Early after injury, patients employ a stiffened knee strategy by decreasing the motion at the knee and co-contracting the scaffolding musculature in a probable effort to support the injured knee against repeated instability while doing normal activities [47,48]. These maladaptations continue despite ACLR [3,4,19,49]. The compensatory movement acts of patients with ACLR are not limited to the rebuilt limb. While doing low-demand activities, such as gait, and high-demanding activities like bilateral jump landings, patients with ACLR change their biomechanical demands from the injured limb to their uninjured limb [3,4,50,51]. The compensatory strategies found between the limbs of the patients with ACLR imply that a unilateral ACL injury can extract a bilateral kinetic response [45] through which injured patients also employ another strategy for the uninjured limb. This may be another manifestation of the aberrant contralateral limb loading patterns employed by a number of patients after surgery [6,46], which could put the contralateral ACL at a higher risk for injury [52]. Overall, the observed biomechanical deviations, particularly during repetitive tasks such as walking, contribute to understanding the chronic changes after ACL surgery.

Our findings also indicate that there is quadriceps strength asymmetry after ACLR. Quadriceps femoris muscle weakness is common after ACLR [15,16]. Studies have examined the relationship between quadriceps strength and gait biomechanics with mixed findings. A positive relationship between quadriceps strength and gait symmetry has been obtained in previous research, suggesting that better quadriceps strength contributes to better symmetry [17,18]. In other studies, however, gait asymmetries have been present despite restoration of quadriceps strength [19,20]. Thus, deficiency in quadriceps strength is not the only predictor of lower-extremity movement asymmetries. Therefore, contextual factors in addition to the functional and muscular performance of the knee after ACLR, such as psychological factors, may be relevant and need to be addressed during recovery and rehabilitation.

Kinesiophobia, which is considered an extreme, irrational, and detrimental fear of physical activity, can occur as a result of sensing susceptibility to injury or reinjury [53]. Fear or anxiety has been documented after ACL injury [21]. Fear of reinjury is one of the most commonly cited reasons for not returning to sport [29,31,54]. In one study, 50% of the athletes who did not RTS after ACLR cited fear of reinjury as a factor for not returning [54]. In another study, athletes who did not RTS after ACLR had higher TSK-11 scores than those who did RTS at six months and one year after ACLR [23]. Although the patients in the current study were approximately six months removed from surgery, a relationship between fear of reinjury and gait asymmetry was observed. Patients may sense that special movements exposed them to the risk of injury early in rehabilitation. Thus, they might anticipate risk and eschew such positions of conceived harm by avoiding tasks or limiting motion to support the body limb [55]. The patients in our study did this by increasing muscle activity and decreasing vGRF in their injured limb, which could have been caused by cautious movement patterns due to fear of reinjury. These guarded movement patterns may have lasting consequences, because they have been reported to persist for years. It should be noted that a similar association between fear of reinjury and protected movement and activation patterns has been reported in patients with elevated fear of reinjury two years after surgery [25]. In their study on the relationship between fear of reinjury and jump-landing biomechanics and muscle activation, Trigsted et al. found that individuals who reported higher fear of reinjury demonstrated enhanced preparatory muscle activation of the quadriceps and gluteus maximus during a bilateral drop vertical jump task [25]. In a study of the relationship between psychological readiness and knee kinematic asymmetry during gait, Zarzycki et al. found that psychological readiness was inversely related to interlimb asymmetry in knee flexion [20].

The current findings join those of other studies in which the TSK-11 (and its 17-item predecessor) has been administered in documenting inverse relationships between fear of reinjury and a wide variety of post-ACLR outcomes [21,23,25,27,30,31,32]. More broadly, the findings dovetail with those of: (a) a clinical review in which it was concluded that along with high levels of psychological readiness and favorable subjective assessments of knee function, low levels of fear of reinjury were associated with returning to preinjury level of sport after ACLR [29]; and (b) a study in which Paterno et al. found that patients who went on to sustain a second ACL injury within the ipsilateral limb had reported significantly greater fear of reinjury prior to returning to sport than patients who did not sustain another ACL injury [32].

The findings of the study have implications for post-ACLR rehabilitation programs. Regardless of movement asymmetries, getting back to sport after 6 months of surgery may expose the athletes to an enhanced risk for reinjury [3], especially if psychological impairments also exist. Lower-limb asymmetry has been shown to predict reinjury of the ACL [6,8,52]. The findings of the present study showed that fear of reinjury was positively associated with asymmetry of muscle activity and vGRF in individuals with ACLR. These data provide potential support for the importance of evaluating and potentially addressing fear of reinjury early in rehabilitation. It can be argued that parallel consideration of psychological factors and movement symmetry should be incorporated into the process of assessing the readiness of athletes to RTS and become exposed to the risk of second ACL injuries. As psychological differences among athletes with implications for RTS outcomes can emerge within six months of ACLR [56], clinicians should consider psychological factors early to assist the patients with modifying their abnormal movement patterns, enhancing their readiness to RTS, and decreasing their risk of incurring a second ACL injury. Movement strategies are correctible and depict significant goals for helpful clinical amendments. Initial assessment and identification of maladaptive or dysfunctional psychological responses during rehabilitation may allow the clinician to address these correctable deficits with targeted interventions prior to RTS. Just as stress management interventions have been found useful in the prevention of sport injury [57,58], psychological interventions that reduce reinjury anxiety (e.g., relaxation and guided imagery) [59] may prove useful in decreasing lower-limb asymmetry and, consequently, reducing the occurrence of ACL reinjury. Future work should build on these preliminary findings to facilitate a better understanding of the role of patient-reported fear and other psychological factors in reinjury of the ACL.

Several limitations of the current study should be considered when interpreting the findings and planning future research. First, the small sample size and all-male cohort make it difficult to generalize the results. Subsequent studies should feature larger samples and include both men and women as participants, as psychological factors may influence lower-extremity movement asymmetry between the sexes differentially. Second, the cross-sectional design prevents drawing causal interpretations from the findings. Longitudinal research is needed to identify the temporal sequence in which kinesiophobia and gait asymmetry develop after ACLR, and experimental studies are needed to determine the extent to which changes in either kinesiophobia or gait asymmetry produce corresponding changes in the other variable.

In addition to addressing the limitations of the current study, investigators can expand the relevance and rigor of the current study by conducting kinematic examination of joint movements. Because quadriceps strength asymmetry can affect symmetrical lower-limb movement, the relationship between psychological factors and gait asymmetry may differ between groups with symmetric and asymmetric quadriceps strength. Further, inclusion of other key ACLR outcomes such as psychological readiness, RTS, knee functioning, subjective knee symptoms, and reinjury is warranted to place the current findings in the broader context of rehabilitation and recovery after ACLR.

## 5. Conclusions

Results revealed a significant relationship between kinesiophobia and asymmetry in muscle activity and vGRF in different phases of the gait cycle. Addressing kinesiophobia early in the rehabilitation after ACLR may support the restoration of gait asymmetry, facilitate a more rapid RTS, and reduce the risk of ACL reinjury.

## Figures and Tables

**Table 1:** Mean and standard deviation of vertical ground reaction force (vGRF) and muscle activity.

		Injured	Uninjured
vGRF (%BW)	First peak	109.94 ± 9.36	102.51 ± 8.80
	Second peak	97.63 ± 6.75	116.37 ± 10.45
Muscle activity in contact phase (%MVC)	Medial gastrocnemius	13.42 ± 3.55	12.37 ± 3.18
	Rectus femoris	34.78 ± 6.24	27.68 ± 5.02
	Biceps femoris	26.57 ± 4.27	22.24 ± 4.02
Muscle activity in midstance/propulsion phase (%MVC)	Medial gastrocnemius	32.27 ± 5.19	35.77 ± 6.05
	Rectus femoris	16.21 ± 3.81	14.43 ± 3.27
	Biceps femoris	12.03 ± 3.12	10.96 ± 2.69

**Table 2:** Correlations between TSK-11 scores and asymmetry of vGRF during gait.

Asymmetry of vGRF (%)	Mean ± SD	TSK-11 Score		Strength of Relationship
		*p* Value	Correlation Coefficient	
First peak	6.98 ± 5.45	0.153	0.200	Low
Second peak	−17.34 ± 7.21	0.002 *	0.531	High

* Denotes a significant relationship.

**Table 3:** Correlations between TSK-11 scores and asymmetry of muscle activity during gait.

Asymmetry of Muscle Activity (%)		Mean ± SD	TSK-11 Score		Strength of Relationship
			*p* Value	Correlation Coefficient	
Contact phase	Medial gastrocnemius	7.81 ± 13.62	0.391	−0.055	Trivial
	Rectus femoris	22.75 ± 4.92	0.007 *	0.460	Moderate
	Biceps femoris	18.02 ± 8.18	0.011 *	0.429	Moderate
Midstance/ propulsion phase	Medial gastrocnemius	−10.13 ± 11.76	0.212	0.157	Low
	Rectus femoris	11.34 ± 5.35	0.098	0.252	Low
	Biceps femoris	8.97 ± 7.15	0.166	0.191	Low

* Denotes a significant relationship.

## Data Availability

The data presented in this study are available on request from the second author.
